# Evaluation of Effective Factors on the Clinical Performance of General Surgeons in Tehran University of Medical Science, 2015

**DOI:** 10.5539/gjhs.v8n9p66

**Published:** 2015-10-29

**Authors:** Fereshteh Farzianpour, Efat Mohamadi, Zhila najafpour, Taraneh Yousefinezhadi, Sara Forootan, Abbas Rahimi Foroushani

**Affiliations:** 1 Department of Health Management and Economics, School of Public Health, Tehran University of Medical Sciences, Tehran, Iran; 2 Departments of Epidemiology and Biostatistics, School of Public Health, Tehran University of Medical Sciences, Tehran, Iran

**Keywords:** evaluation, effective factors, clinical performance, general surgeons, Tehran University of Medical Science

## Abstract

**Background and Objective:**

Existence of doctors with high performance is one of the necessary conditions to provide high quality services. There are different motivations, which could affect their performance. Recognizing Factors which effect the performance of doctors as an effective force in health care centers is necessary. The aim of this article was evaluate the effective factors which influence on clinical performance of general surgery of Tehran University of Medical Sciences in 2015.

**Methods:**

This is a cross-sectional qualitative-quantitative study. This research conducted in 3 phases-phases I: (use of library studies and databases to collect data), phase II: localization of detected factors in first phase by using the Delphi technique and phase III: prioritizing the affecting factors on performance of doctors by using qualitative interviews.

**Results:**

12 articles were analyzed from 300 abstracts during the evaluation process. The output of assessment identified 23 factors was sent to surgeons and their assistants for obtaining their opinions. Quantitative analysis of the findings showed that “work qualification” (86.1%) and “managers and supervisors style” (50%) have respectively the most and the least impact on the performance of doctors. Finally 18 effective factors were identified and prioritized in the performance of general surgeons.

**Conclusion:**

The results showed that motivation and performance is not a single operating parameter and it depends on several factors according to cultural background. Therefore it is necessary to design, implementation and monitoring based on key determinants of effective interventions due to cultural background.

## 1. Background

Human resources are intellectual and capable factors that can create powerful and dynamic organization by optimizing the use of other resources (Armstrong et al., 2010). Obviously motivated human resources can change everything in their working environment to the benefit of the community by power of fortitude and valuable experiences (Armstrong et al., 2010). Satisfaction of employees is a very important issue in human resources and considering it has an undeniable impact on the performance of the organizations’ staff (Armstrong et al., 2010). As long as the staff have unacceptable satisfaction, motivation, and job commitment, other activities of the organization will not have the necessary result and consequence (Burtson Paige et al., 2010). Job Motivating and directing and healthy incentive creator to use are one of the tools of human resources. If a person is motivated, he can be creative and self-command in his job (Muhdi Louise et al., 2011). Therefore, understanding the factors that cause job motivation and upgrade the performance is one of the necessities that can be involved in the development of productivity and job satisfaction (Danish et al., 2010). The success of an organization is determined by the employees’ decisions and behaviors that they are encouraged to. Therefore, a vital resource for the success and development of the organization is having appropriate systems for the taking, keeping, motivating, and developing organization’s human resource. The role of human resources in today’s world has increased the importance of human resource management several times with the use of specialized and trained resources (Danish et al., 2010; Martineau, 2003; Marce Jeger, 2002; David Mcutler, 2006; Kesteloot et al., 1998; Decker, 2012; Salvendy, 2012; Hendry, 2012; Dowling et al., 2008). One of the important tasks of managers in organizations is to identify potential talents of employees and providing areas of growth and development potential and providing them the context of realizing the objective of a major improvement for them that provides the background for the important and fundamental aim of productivity improvement. In other words, knowledge of the issues motivating employees is very important to improve performance and increase the productivity of an organization. Also, gaining such knowledge can profoundly help functional improvement of organization’s human resources and help in preventing employees’ resistance to change, reducing restrictions on efficiency and staff’s fighting with each other that result in creating a profitable organization (Bratton et al., 2012). This allows and forces administrators to form the internal and external environment of the organization in such a way to select proper goals to satisfy the needs of employees and be successful in motivating the staff (Storey et al., 2014). Health system, as a trustee of people’s health, has the most diverse human resources with quite different combinations. One of the most important and most influential of them are doctors. Of the necessary conditions to provide high-quality services are empowered physicians with a high level of performance. Different motivations can affect their performance.

## 2. Methods

This cross-sectional quantity-quality study research was conducted in three phases. The first phase, research: In this phase, information on performance evaluation, motivational factors of improving performance, and factors affecting the performance of doctors was collected using databases including The National Library of Medicine, Pub Med, EMBASE, Science Direct, Scopus, Springer link, and Google Scholar.

The second phase, indigenousness of the identified factors: In this step, using the results of the first stage, the Delphi technique was used to criticize and validate initial list, the identified factors affecting the operation of general surgeons were presented to participants (doctors, general surgeons, and treatment deputies) and their feedback was collected.

The third phase, prioritizing the factors affecting the performance of physicians: In the final stage of the project, researchers discussed the factors affecting the performance of the doctors in focused group sessions and ranked these factors in order of their depth of effect.

## 3. Results

The findings are adjusted in four sectors including: the results of the comprehensive review of studies, quantitative analysis of questionnaires, qualitative analysis of interviews, and summarizing the findings of the study.

**The findings of the comprehensive review of studies**

In this phase of the study, the most important keywords and databases, including Google Scholar, Pub Med, Pro Quest, Cochran library, ISI web of knowledge, Scopus, and Embase (1990 to present) were studied.

The study began with a literature review of 300 abstracts. After inspection, 154 abstracts were excluded because of remoteness from the subject and 146 articles were evaluated. The assessment output was 12 articles. The information of 12 articles were evaluated in “general information” and “specific information” aspects. General information included: title, first author, year of publication, the performer country, the country of field of study, the journal, and the aim; and specific information included: author, type of study, method of data collection, data analysis, and findings. After assessing and analyzing the articles, identified themes were extracted and factors affecting employees’ performance were listed case by case.

**Table 1:** Themes extracted from studies evaluating the factors affecting employee’s performance

Identified themes affecting employee’s performance	Study’s title
Expectations, job values, personal job qualities, psychological characteristics of individuals, organizational culture, organizational characteristics, job characteristics, and cognitive factors	Determinants and consequences of motivation of health workers in hospitals in Jordan and Georgia

Factors associated with staff’s health (knowledge, skill, job satisfaction, job experience, fear of ill-treatment result, and job commitment)	How can we achieve high-quality performance of health staff using little resources
Patient-related factors (severity of disease, social determinants of patients’ health)	
Job characteristics (complexity and transparency of guidelines, and change in the guidelines)	
The environment of providing service (work processes quality percent, management and central leadership, colleagues’ communication, access to equipment and supplies, and private or government center)	
Professional environment, scientific environment, sociocultural environment, economic environment, and political environment	

Access to information, characteristics of the job environment, and organizational culture	Innovation and motivation in public health professionals

Job satisfaction, organizational commitment, motivation of service delivery, and organizational citizenship behavior	Individual factors and organizational performance in government agencies

Financial factors, career development, continuous education, hospital infrastructure, resources availability, hospital management, and personal understanding and knowledge	Motivation and preservation of health workers in developing countries

Job skills, leadership and management practices, ground for promoting employment, and availability of job facilities	Improving motivation of primary health care staff in Tanzania; health staff approach

Encouraging and rewarding, proper job environment, reimbursement system, education, availability of devices and equipment, being recognized by supervisors, and the nature of the job	Motivation of health staff in Africa, assessing non-financial motivational factors and human resource management tools

Individual characteristics (sex, age, marital status, etc.)	High ability to work and function in mental health services: organizational, individual, and background factors
Personal resources (flexibility, optimism, pessimism, and self-efficacy)	
Job requirements (quantities, emotional, and cognitive needs)	
Career resources (influence at work, opportunities for development, a degree of freedom at work, community sense, feedback, leadership quality, social protection, and social relations)	
Correlation in job (power, dedication, and absorption)	

Manager’s support, colleagues’ respect and acceptance, income, job security, and job interest	Identifying factors affecting job motivation in health staff in northern Vietnam village
Job requirements(quantities, emotional, and cognitive needs)	Surgeons’ job cooperation: affecting factors and associates to job and life satisfaction
Career resources (influence at work, opportunities for development, a degree of freedom at work, community sense, feedback, leadership quality, social protection, and social relations)	
Correlation in job (power, dedication, and absorption)	

Negative factors affecting performance(gender, personality type A, lack of control, and lack of social support)	Review and prioritization of stressors affecting job performance in administrative staff of *Orumieh* University of Medical Sciences
Occupational factors affecting the performance negatively (role overload, role shortage, role conflict, and role ambiguity)	
Organizational factors affecting the performance negatively (wage policies, job insecurity, changing job’s place, and technology)	

Income and salary, job position, job environment conditions, professional development, nature of work, and job contacts)	Checking the status of the motivational factors affecting the staff performance in North Khorasan University of Medical Sciences

The primary identified themes were divided into three categories: personal factors, job and its nature, and organizational factors and job environment. The output was provided to general surgeons as the table below.

**Table 2:** Evaluated components

Evaluated component	
**Personal factors**	1. Competence to perform the job
	2. Interest in job
	3. Person’s knowledge of the job and its importance
	4. Personality type
	5. Age
	6. Gender
	7. Job background (experience)

**Job and its nature**	8. The tariffs
	9. The payment method (income, salary, etc.)
	10. The total amount of salary
	11. Freedom degree at job
	12. Opportunity of development and scientific progress
	13. Date of scientifically
	14. Social status (reputation, fame, etc.)
	15. Job security (employment type)
	16. Diversity in job
	17. Professional responsibility
	18. The role ambiguity (academic degree)
	19. Job’s impact (believing in job influence)

**Organizational factors and job environment**	20. Professional communications
	21. How to manage and care (hospital, group)
	22. Access to technology and tools needed to do the job
	23. Access to supplemental human resources required to do the job

**Analysis of demographic variables**

In the current study, 22% of the samples aged 30 to 39 years, 38.9% of them aged 40 to 49 years, 5.6% of them aged between 50 and 59 years, and 5.6% were over 60 years old. 33.3% of the participants were female, 66.7% were men. Minimum job experience was 2 years and maximum was 20 years. 25% of the samples had less than 5 years of job experience, 81% of them less than 10 years, 92% less than 15 years, and 100% of them less than 20 years.

**The output of the first stage of Delphi**

After the implementation of the first phase of Delphi, the results were determined as follows. Given that more than 50% had over 60% consensual opinions, the following consensus was achieved in the first phase of Delphi.

**Table 3:** Factors affecting performance scores respective to the highest score

Score level	Title		Amount (percentage)
Range between (75%-100%) maximum effect	1	Competence to perform the job	**(86.1%)**
	2	Interest in job	**(83.3%)**
	3	Person’s knowledge of the job and its importance	**(80.6%)**
	4	Personality type	**(80.6%)**
	5	Age	**(77.8%)**
	6	Gender	**(77.8%)**
	7	Job background (experience)	**(77.8%)**
	8	The tariffs	**75%**
	9	The payment method (income, salary, etc.)	**75%**
	10	The total amount of salary	**75%**
	11	Freedom degree at job	**75%**

Range between (55%-74%) medium effect	12	Opportunity of development and scientific progress	**(69.4%)**
	13	Date of scientifically	**(69.4%)**
	14	Social status (reputation, fame, etc.)	**(69.4%)**
	15	Job security (employment type)	**(66.7%)**
	16	Diversity in job	**(58.3%)**
	17	Professional responsibility	**(58.3%)**
	18	The role ambiguity (academic degree)	**(55.6%)**
	19	Job’s impact (believing in job influence)	**(55.6%)**

Range between (0%-54%) minimum effect	20	Professional communications	**(52.8%)**
	21	How to manage and care (hospital, group)	**(52.8%)**
	22	Access to technology and tools needed to do the job	**50%**
	23	Access to supplement human resource required to do the job	**50%**

**Qualitative analysis of interviews**

In this study, with implementation of Delphi, an open question was asked from participants to complete and correct the identified factors. In this part, we analyzed the conducted interviews qualitatively. The conceptual framework in the process of qualitative analysis was corrected, if necessary, and some of the subgroups of concept changed in this process. Finally, 3 topics and 11 sub-topics were identified in examining the relationship between the insurer and hospitals.

**Table 4:** Key topics and sub-topics of the factors affecting the performance of general practitioners

Key topics	Sub-topics
Topic1: The effect of individual factors on performance	Factors influencing psychological conditions
	Type of view
	Professional responsibilities
	Job ethics
Topic2: The effect of Environmental factors on performance	Environmental and job conditions
	Facilities of surgeons
	Quality and quantity of complementary human resources
	Job security
	Kind of surgeons’ communication
Topic3: The effect of political factors on performance	Reducing the gap between private and public hospitals Medical ethics

In the final phase of the study, the results of the implementation of the Delphi techniques and analysis of interviews led to identify factors affecting function of general surgeons. In this section, these factors are presented in a table.

**Table 5:** Final table of the factors affecting general surgeons’ performance

Number	Identified factors
1	Competence to perform the job
2	Interest in job
3	Person’s knowledge of the job and its importance
4	The payment method (income, salary, etc.)
5	Freedom degree at job
6	The role ambiguity (academic degree)
7	Professional responsibility
8	Personality type
9	Age
10	Gender
11	Job background (experience)
12	Tariffs
13	The total payment (income, salary, etc.)
14	Opportunity of development and scientific progress
15	Job security (employment type)
16	Type of view
17	Environmental and facility factors
18	Medical ethics

## 4. Discussion

The health and treatment sector account for a large part of costs and doctors has a very significant role, so their performance is evident on health sequences of the country. The results of the current study showed the role of factors of competence, passion, experience, and knowledge of the person from the job and salary payment method on general surgeons’ performance.

Competence to do the job, interest, and understanding of the job have been identified as the most influential factors on performance of physicians. Similar to the study of Franco and et al. (2005) that identified the risk factors and consequences of motivation of health workers in hospitals in Jordan and Georgia, including the Competence to do the job, expectation, job values, job’s personal characteristics, psychological characteristics of individuals, organizational culture, organizational characteristics, job characteristics, and cognitive factors. In the study by Mache et al. (2015), Competence to do the job and job interaction was identified important in psychologists’ performance. Significant correlation was found between practitioners ‘resources of the interaction, the ability to work, and organizational factors (such as opportunity of development, income, etc.). In addition, significant differences were observed in sex, age, and marital status with the ability to work. Another study also reported a significant correlation between the surgeons’ job and job commitment, job satisfaction, and quality of life. In addition, there was an association between job contribution by the relationship between organizational factors and surgeons ‘satisfaction (Mache et al., 2015).

Method of payment ranked fourth, which was among factors with a score high. Muhdi and et al. also state that the payment method can potentially affect the performance (Muhdi et al., 2011). Marce and et al. showed in the study of financial incentives of physicians in health care management that payment based on performance causes an increase in doctors’ visits (Marce et al., 2006) and Rezazadeh (Reza Zadeh et al., 2013) reported that the majority of staff considered the salary and job environments’ conditions as the most important factors affecting their performance. There was also a significant relationship between the job’s location and the employment status with some of the motivational factors. Thus, it is proposed to implement performance-based payment with creating appropriate infrastructures.

Personal characteristics such as age, sex, and type of personality were the factors affecting physicians ‘motivation. Four factors, including factors affecting psychological condition, type of view, professional responsibility, and job conscientious, were the most important items reported by interviewees in this area. These factors have been emphasized in Heydari’s study (Heidari, 2014) and the results showed that occupational, individual, and organizational stressors had the highest impact on job performance.

Another factor affecting doctors’ performance is job security and development that were among factors with high priority and interviewees also reported its significance^2^. One of the participants said in this regard: “I suppose, job security should be evident. As too much or too little of it can be both damaging and harmful” (Interview 12), which is similar to Heydari’s results, conducted on prioritization of stressors affecting job performance of staff of Uremia University of Medical Sciences, reported negative factors affecting the performance (role overload, role shortage, role conflict, and role ambiguity), organizational factors affecting the performance negatively (wage policies, job insecurity, changing job’s place, and technology). Special jobs induce boredom and disgust after a while and weakens the job motivation in individuals that can be overcome by diversifying jobs, promoting employment, and providing further education (Ranjbar et al., 2006). Nevertheless, Hazavehei’s study showed that the highest score among the motivating factors included the availability of conditions, job security, and intrinsic interest to teach that is consistent with the results of the current study (Hazavehei et al., 2007).

The results of Hayes Bronwyn and et al. (Hayes Bronwyn et al., 2010) also indicated that career development, education simultaneous to work, and access to sources are effective motivators, which is consistent to the results of the current study on the role of job development in motivation and promotion of physicians performance, especially the faculty.

Other factors affecting the physicians’ performance are the gap between private and public hospitals and medical ethics, which included the following according to the conducted interviews: factors such as differences in services’ tariff, freedom, the possibility of promotion, and career development. One of the interviewees said: “Unfortunately, the increase in tariffs causes an increase in the quantity reduction and will probably result in quality reduction in government hospitals” (interview 8).

In study of Dowling et al. (2008), the results indicated that motivation is influenced by both financial and non-financial incentives. The main motivating factors for health workers include being appreciated by managers, colleagues and society, a stable job and income, and training. The main discouraging factors included low salaries and difficult job conditions. The results of study of Wright and et al. (2010) indicated motivation variables, feedback, and organizational support as the greatest effectors on productivity and performance of the staff.

The findings show the effect of variable intrinsic factors on physicians’ performance and study Manongi and et al. (2006) also considered intrinsic factors important in creating motivation, which is consistent to the results of the current study. In the study, the environmental factors affecting general surgeons’ function included access to complementary human resources required to perform the job, access to technology and tools needed to do the job, how to manage and supervise (hospital, group), and job communications. However, the factors mentioned contradict the factors reported by one of interviewees of this study that generalized the environmental factors to job conditions, facilities of surgeons, quality and quantity of complementary human resources, job security, and communication between surgeons. One of the interviewees said: “The impact of environmental factors must also be considered psychological conditions” (interview 14). Another doctor said: “In general, factors related to environmental conditions affect health staff, especially physicians. In addition to stressful environment, occupational factors may also play a role, which all in all affects the surgeons’ performance” (interview 18). These results similar to Kim’s study (Kim, 2005) that indicated that employee’s satisfaction, interpersonal communication, and public services motivation affect the employee’s performance and organizational performance.

Mathieu’s study and et al. (Mathauer et al., 2006) also considered moral and ethical aspects effective on employee’s motivation. However, in this study, interviewees ranked occupational ethics 18th in their performance. While weakness in occupational ethics causes reduction in communications and increasing the risk of patients’ injury and can profoundly affect the activities and results of treatment, resulting in enhanced productivity and improved communication. Therefore, it is suggested that the health system consider the implementation and promotion of the rule of professional ethics more seriously, which subsequently reduces stress dramatically and improve performance. Creating a work environment, in which employees work with patients and other members of the treatment group in peace of mind and without stress is effective on improving professional ethics.

The results showed that physicians’ motivation and function is not a single operating parameter and several factors play a role with regard to cultural background. Therefore, it is necessary to implement and monitor more effective interventions based on key determinants or cultural background design. The health authorities should pay more attention to the importance and role of factors affecting job motivation. Individual factors, such as the competence to do the job, interest in the job, person’s knowledge of the job and its importance, personality type, age, and work background (experience); and organizational factors, such as the tariff, payment method (salary, wage, etc.), possibility of development and scientific progress, job security (employment type), diversity in the job, professional responsibility, and job’s professional relationships are the most important effectors of performance. Thus, health authorities should pay attention to the role of these factors and plan long-term to improve motivation and consequently the performance of physicians and prevent them from leaving the system and the country.

**Contributors**

FF contributed to the conception and design of the work, and the acquisition, analysis and interpretation of data, and drafted the paper. EM, JN, TY, SFandARFcontributed to the conception and design of the work, and the acquisition, analysis and interpretation of data. EM contributed to the conception and design of the work, and the acquisition and interpretation of data. All authors contributed to the revision of the paper and approved the final manuscript for publication. All authors have agreed to be accountable for all aspects of the work in ensuring that questions related to the accuracy or integrity of any part of the work are appropriately investigated and resolved.
